# Anthropogenic food resources sustain wolves in conflict scenarios of Western Iran

**DOI:** 10.1371/journal.pone.0218345

**Published:** 2019-06-17

**Authors:** Alireza Mohammadi, Mohammad Kaboli, Víctor Sazatornil, José Vicente López-Bao

**Affiliations:** 1 Department of Environmental Sciences, Faculty of Natural Resources, University of Tehran, Karaj, Iran; 2 Department of Environment Sciences, Faculty of Natural Resources, University of Jiroft, Jiroft, Iran; 3 Forest Science and Technology Centre of Catalonia (CTFC), Solsona, Spain; 4 Research Unit of Biodiversity (UO/CSIC/PA), Oviedo University, Mieres, Spain; University of Sydney, AUSTRALIA

## Abstract

The feeding ecology of gray wolves has been investigated extensively worldwide. Despite previous studies on food habits of wolves in Asia and Iran, none has focused on the diet of the species in a scenario of depleted of wild prey and with recent records of attacks on humans. Here, we combined telemetry methods and scat analysis to study the diet of wolves in areas of Hamadan province, Iran, where medium to large wild prey is almost absent. Between October 2015 and March 2017, we studied the feeding behavior (by identifying feeding sites through clusters of GPS locations) of three wolves fitted with GPS collars, belonging to different wolf packs. We also collected and analyzed 110 wolf scats during the same period within the same areas. Overall, we investigated 850 clusters of GPS locations in the field, and identified 312 feeding sites. Most feeding clusters were linked to dumpsites and poultry farms around villages. We found 142 and 170 events of predatory (kill sites) and scavenging behavior, respectively. Prey composition based on kill sites was comprised of 74.6% livestock, 19.7% lagomorphs, 3.5% dogs, 1.4% red fox, and 0.7% golden jackal. Similarly, prey composition based on scavenging clusters was comprised of 79.9% livestock, 10.6% red fox, and 9.4% golden jackal. Scat analysis, however, indicated that livestock (34.3%), garbage (23.7%), poultry (16.0%), and European hare (15.4%) were the most frequent food items. We discuss the role of anthropogenic food sources in a context where agonistic wolf-human encounters occur recurrently, and suggest management guidelines regarding illegal dumping of animal carcasses and garbage dumpsites, in order to minimize wolf-human negative interactions.

## Introduction

The predatory behavior of large carnivores may represent one of the main factors triggering large carnivore persecution worldwide, particularly in human-dominated areas [1–8]. It is also well established that food of human origin can become a substantial resource for large carnivores in these landscapes [9–11]; which can adapt their behavior accordingly [12] or rely largely on these resources [13–15].

Shortage of wild prey cumulatively increases carnivores’ reliance on livestock [11, 13–18], which can trigger human-large carnivore conflict situations. Hence, implementation of effective management strategies to mitigate the impact of large carnivores on livestock [19–21] is often required from multiples sectors of the society and contingent upon gaining a deep knowledge of the dietary performance of large carnivore species.

The feeding ecology of wolves *(Canis lupus)* has been extensively studied throughout its worldwide range [22–25]; although information from Asian scenarios is still limited [26–28]. Wolves are adaptable to a wide arrange of food items. According to previous studies, wolves in Asia feed on a wide variety of anthropogenic food items, such as livestock, poultry, garbage, carrion or cultivated fruits [27–35]. This trophic plasticity allows wolves to persist in multiple human-dominated landscapes [11, 36–37] even in scenarios with low abundance of wild prey [14, 38].

In some Asian human-dominated landscapes, such as in western Iran, the low abundance of wild prey, together with the lack of an effective management of organic waste, has been hypothesized as one of the factors behind negative wolf-human interactions, including livestock depredation and events of wolf attacks on humans [39, 40]. For example, in Hamadan Province (western Iran), due to the lack of knowledge on the best practices to minimize the chances of negative wolf-human interactions, the majority of local communities illegally dump their organic waste and livestock carcasses near poultry farms and in their backyards [41]. In a questionnaire survey exploring different human dimensions associated with wolf presence in this area, after interviewing 400 people owning livestock in this province, 63.5% of them followed these practices for the management of livestock carcasses and garbage [Authors, unpub. data].

Despite previous studies on the diet of wolves in Iran [27,28], no evaluation has been done in an area with records of wolf attacks on people; which is also unique from a worldwide perspective. In this regard, in Hamadan Province, oofficial records indicate that, apart from reports of livestock depredation events, about 60 incidents of wolf attacks on people have occurred since 2001 [39,40]. As a consequence, there is an urgent need to understand the mechanisms behind the occurrence of these events, compared to the rare frequency that they occur elsewhere [42–43] in order to delineate effective management strategies to reduce the likelihood of their occurrence.

Several hypotheses have been suggested to explain the observed increment in human-wolf conflicts in this rural area, such as an extremely low abundance of wild prey, habituation to humans (wolves’ tendency to feed closer to human settlements) or different unwanted human behaviours (e.g. practices favouring the availability of waste). However, lack of information about wolf behaviour does not allow testing these hypotheses properly. Assessing the dietary composition of wolves and the spatial distribution of food sources may be a starting point to understand this scenario properly and to delineate effective conflict mitigation measures in an area depleted of wild prey. The use and distribution of anthropogenic food sources may play a significant role in wolf-human interactions and risk assessment [14, 44].

Accordingly, here, we combined information from GPS collared wolves and scat analysis to evaluate the feeding behaviour of wolves. Although the diet of wolves can be evaluated using scat or stomach analysis [14, 23–44], which involves identifying prey remains, Global Positioning System (GPS) satellite collars facilitate the location of kill or feeding sites [45–51]. These approaches complement each other providing fine-scale information about different food items present in the diet of wolves [46–52], the occurrence of predatory and scavenging events, and the effect of different food sources on the spatial ecology of the species.

## Material and methods

### Study area

**Fig 1:**
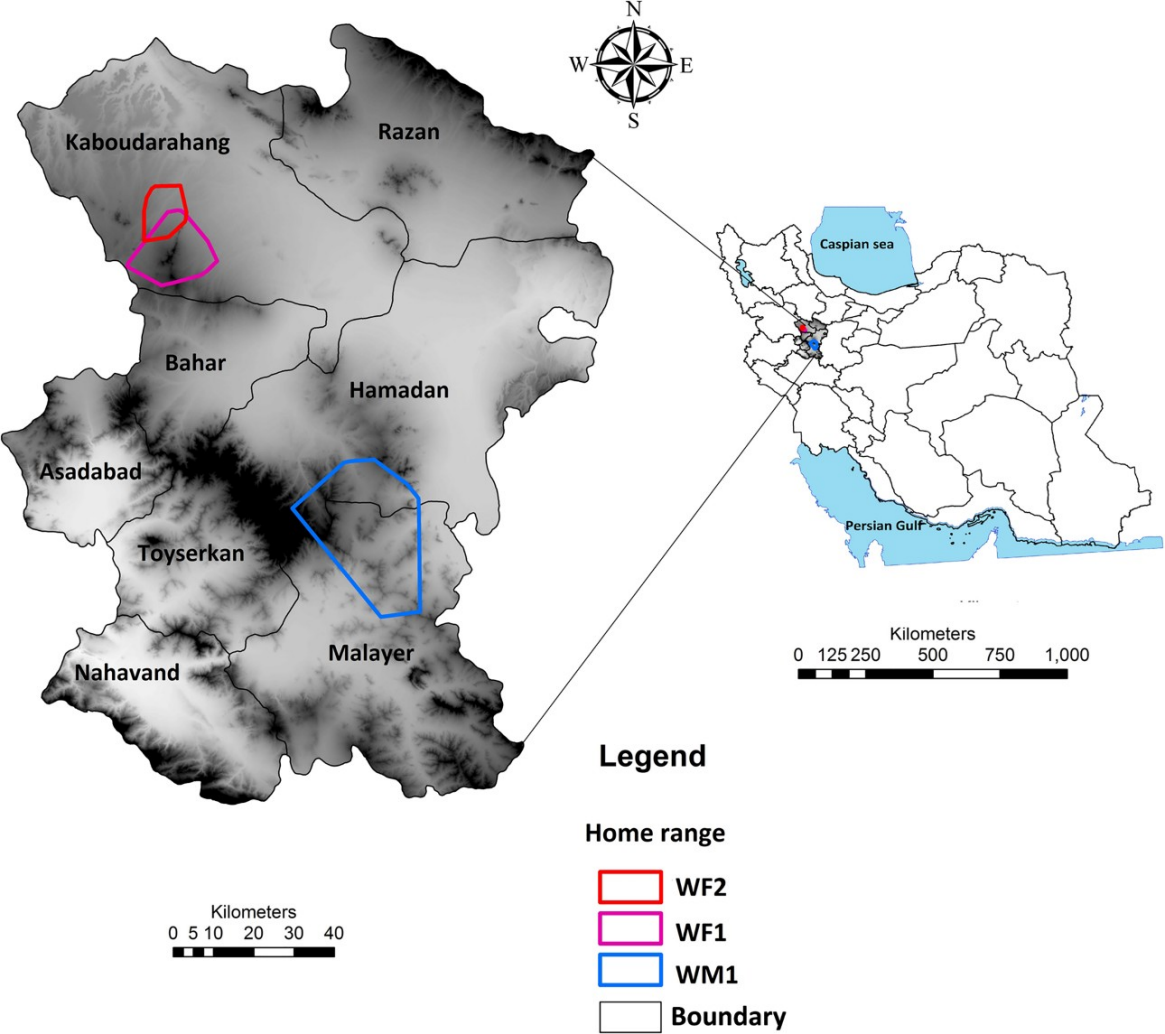
Location of the study area in Hamadan province (34.7608° N, 48.3988° E), west of Iran (DEM map was downloaded from the WorldClim database (www.worldclim.org)) with position (Home range) of tracked wolves.

This study was conducted in Hamadan province, Western Iran (Fig 1), particularly in Alisadr and Hamadan counties. The province encompasses approximately 19,493 km^2^ and supports a population of over two million people. Hamadan province is characterized by a human-dominated landscape with a mean human population density of about 88 inhabitants per km^2^, twice the mean population density of the country [39]. Economic activities in the region mainly consist of livestock rearing and agriculture. Herds of sheep (*Ovis aries*) and goat (*Capra aegagrus hircus*) mostly graze freely in specifically designated rangelands, under the care of shepherds (in many cases including children) and guard dogs (most of them are not trained to deter wolves), and are kept in covered pens at night, either in villages or on rangelands [39].

### Methods

#### Wolf collaring and GPS clusters visits

Between October 2015 and March 2016, we captured three wolves, including two adult females (WF1, WF2) and one adult male (WM1), using Belisle traps. Each wolf was immobilized using a combination of Ketamine (6 mg/kg) and Xylazine (1 mg/kg) [41]. The wolves were evaluated as clinically healthy at the moment of capture. Captures were carried out under permit 94/31147 from the Iranian Department of Environment.

The GPS collars (Iridium version, Followit Tellus, Lindesberg, Sweden) were programmed to record a location every 8 h 20 days per month. However, in order to identify predation (kill sites) and scavenging events, this general schedule was alternated with an intensive schedule designed to obtain a location every 20 min (72 locations/day) [51] for the remaining 10 days of every month. During the intensive schedule periods, we identified clusters of GPS locations, indicating potential feeding sites (i.e., kill or scavenging events). We considered two or more locations with a maximum in-between distance of less than 60 m to identify potential feeding sites to visit in the field [51].

After GPS clusters were identified, we subsequently visited them within 48 h whenever possible (90% of clusters were visited within this time period). All locations identified in a cluster were visited, and in every location, we explored a 30-m radius (based on GPS error) searching for prey remains [51]. Once clusters were evaluated *in situ*, we categorized them as a searching site (wolf activity signs, with no feeding evidence), feeding site (prey remains or other food sources), resting points and unknown (other/no signs). Prey remains in each cluster were photographed, evaluated in order to discriminate between predation and scavenging events (temporal congruence between consumption and carcass condition, presence of wounds compatible with depredation, evidence of road killed animals, etc.) and, if needed, representative material was taken for later identification of the prey item in the laboratory.

To better discriminate between scavenging of domestic animals and depredation events, we also interviewed local people (N = 150) after visiting the feeding clusters. During our study, some of the livestock deaths in rural areas were disease-related. Thus, immediately after locating the feeding clusters around each village, the villagers were interviewed to determine whether the potential preys had been abandoned by local communities or not. Among the 400 interviewees, 300 (75%) reported livestock deaths due to diseases of Foot-and-Mouth fever, whereas 150 interviewees (37.5%) reported wolf depredation on their livestock. The majority of livestock accessible to wolves had died from diseases [Authors, unpub. data].

#### Scat analysis

Within wolf home ranges (Minimum Convex Polygon using 100% of GPS locations), we collected wolf scats both at the GPS clusters and opportunistically (i.e. independent of cluster investigations) [46]. Only samples with a diameter of more than 20 mm were collected for analysis to minimize the collection of non-wolf scat samples (scats were identified based on shape and size) [47–53].

For scat sampling, we attempted to keep the inherent biases of sampling to a minimum in order to avoid false analysis [46]. For instance, to diminish pseudo-replication of independent scats at a kill site, we only collected a single scat at each GPS cluster or random location [46].

Scats were classified belonging to feeding sites, other GPS clusters, or opportunistic findings. All scats were washed using water through a metal sieve (1.5 mm mesh), leaving only undigested prey remains, predominantly hair and bone fragments. Then, from each scat, 20 hairs were randomly chosen and positioned on microscope slides to be later examined under a microscope [46]. Hair identification was done according to the cuticle scale and medulla patterns [46].

We used the frequency of occurrence of the different prey items (being frequency of occurrence fitted to a 100%) and relative biomass [44,54]. To analyze the frequency of occurrence of each prey in the scats, we calculated its occurrence relative to the total prey items identified in the scat [46]. We used this method to account for cases where > 1 prey item was found in a scat. We applied Weaver et al. 1993 [55] correction factor to the occurrence data, Y = 0.439 + 0.008X, where Y is the weight of prey consumed per collectable scat (kg/scat) and X is the mean prey body weight [56] (S1 Table). Assuming that one small prey item does not comprise a total scat, we did not apply the correction factor to prey items < 2 kg [46]. To calculate the biomass of each prey consumed, the correction factor for that prey item was multiplied by the occurrence of the given prey item relative to all prey items. Next, to measure the relative amount of biomass that was consumed of each prey, we divided the biomass consumed of each prey by the total biomass consumed. Percentage of carcasses eaten was calculated by dividing the number of carcasses eaten by the total number of carcasses eaten, multiplied by 100.

We used G-tests (independent t-test) to determine whether estimated prey composition was similar for ‘GPS cluster analyses’ versus ‘scat analysis’ (using all scats found) [53].

#### Spatial feeding behavior of wolves

In order to determine the spatial relationship between predation and scavenging sites, and human settlements, we compared the distance (m) to the closest human settlement between the observed feeding clusters and a set of random locations (N = 312) within wolf home ranges. We used Wilcoxon signed–rank test to determine whether wolves had a tendency to feed closer to human settlements than randomly expected. Furthermore, we created a 1 km circular buffer around each feeding site, human settlement and dumpsite within wolf home ranges as well as the location of reported wolf attacks on humans since 2001 [39], in order to evaluate the existence of spatial overlap between feeding clusters and the location of attacks, dumpsites and human settlements. To do this, we evaluated the spatial overlap among the different buffers using Arc GIS Version 10.4 (ESRI, Redlands, CA). We used the locations of confirmed wolf attacks on humans based on the previous work by Behdarvand and Kaboli [2015], as well as additional data on wolf attacks provided by the Hamadan Department of Environment (HDOE). The location of human settlements and dumpsites within wolf home ranges was obtained from the HDOE. Statistical procedures were run using Arc GIS software.

## Results

### GPS clusters

**Fig 2:**
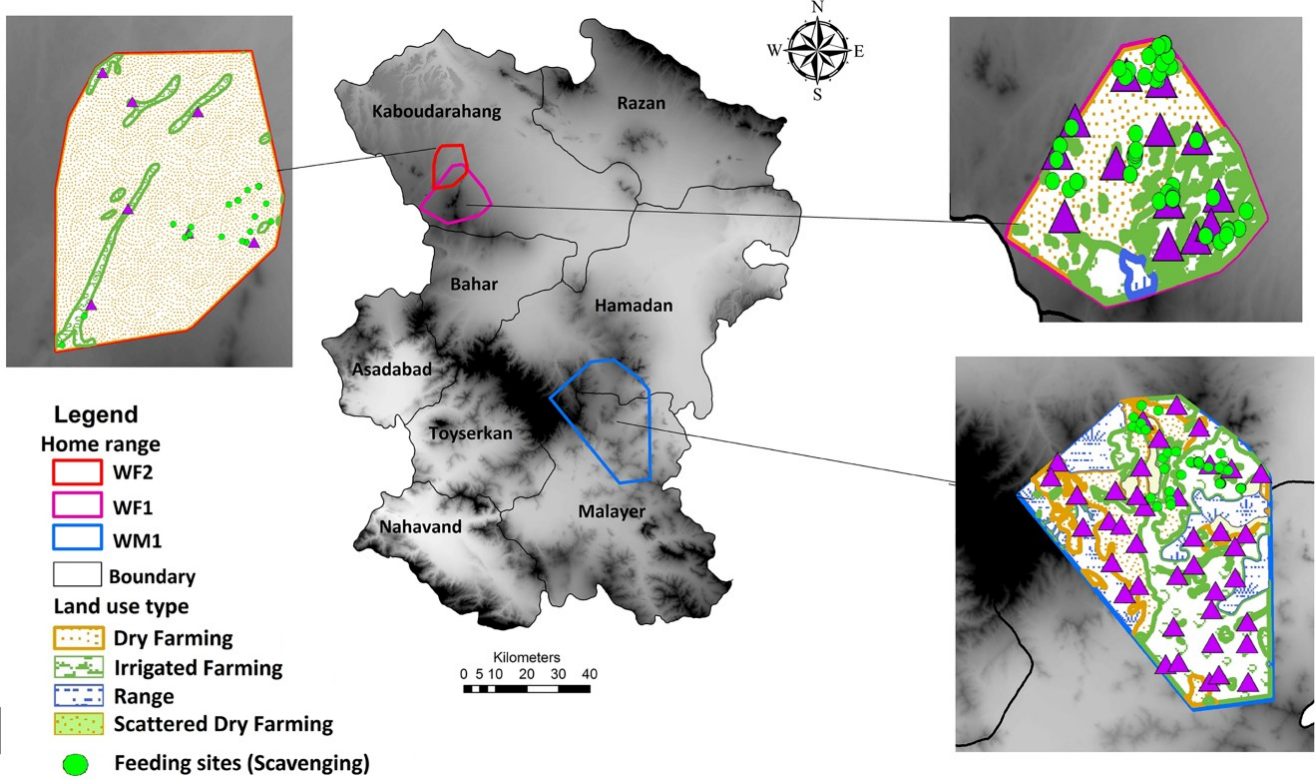
Scavenging of domestic animals by tracked wolves (WM1, WF1 and WF2) near houses and farmlands in Hamadan province (DEM map was downloaded from the WorldClim database (www.worldclim.org)).

Between October 2015 and March 2017, we investigated a total of 827 GPS clusters in the field. Overall, we located 312 feeding sites (Table 1). Importantly, most feeding sites (57.6%) as well as searching sites (50.6% out of 425 searching sites in total), were located around human settlements, dumpsites and poultry farms (less than 1 km) (Table 1). Among the 312 clusters of feeding sites, 142 events were associated with predatory behavior (kill sites) and 170 clusters were associated with scavenging behavior (46% predation and 54% scavenging events) (Table 1). Scavenging of domestic animals occurred near houses and farmlands (dry farming and irrigated farming) where livestock carcasses that died by causes other than wolves were disposed by local people (Fig 2). On the other hand, scavenging of wildlife was recorded near roads, where animals such as red foxe *(Vulpes vulpes)* and Golden jackal *(Canis aureus)* were road-killed (Table 2).

**Table 1:** Number of GPS clusters investigated per wolf in Hamadan and Alisadr counties. Clusters were categorized into feeding sites (predation and scavenging sites), resting sites, searching sites, and unknown.

			Feeding site		Searching site	Unknown
Individual	Number of cluster investigated	Rest	Predation	Scavenging	Near poultry and in dumpsite	
WF1	330	10	40	70	200	10
WM1	400	30	90	95	155	30
WF2	97	5	12	5	70	5
Total	827	45	142	170	425	45

**Table 2:** Feeding remains located using GPS clusters in kill and scavenging sites.

	kill sites (142)					scavenging sites (170)			
Prey type	Estimated mean weight of prey (kg)	N. of kills	% of kills	Biomass consumed (kg)	Biomass consumed as % of all kill sites	No. of carcass eaten	% of carcass eaten	Biomass consumed (kg)	Biomass consumed as % of all kill sites
Livestock (sheep)	25	106	74.6	2650	91.09	115	67.6	2875	22.8
Livestock (cattle)	450	0	0	0	0	21	12.3	9450	75.0
European hare	3.5	28	19.7	98	3.36	0	0	0	0
Golden jackal	11	1	0.7	11	0.37	16	9.4	176	1.4
Red fox	5	2	1.4	10	0.34	18	10.6	90	0.7
Dog	28	5	3.5	140	4.81	0	0	0	0
Total	553.5	142	100	2909	99.97	170	100	12591	99.9

At kill sites (WF1: n = 40, WM1: n = 90, WF2: n = 12), we identified prey items from five group of species. On the other hand, at scavenging sites (WF1: n = 70, WM1: n = 95, WF2: n = 5), remains of three group of species were found. Domestic animals accounted for 74.6% of prey items identified at kill sites, and this figure was similar at scavenging sites (79.9%) (Table 2). The only wild prey species found at GPS clusters were European hare (*Lepus europaeus*) (19.7%), golden jackal (0.7%) and red fox (1.4%) (Table 2).

The majority of biomass consumed was livestock, both in GPS clusters and scats (details of feeding remains located using GPS clusters for each collared wolf is provided in S2–S4 Tables). In the scavenging sites, the majority of biomass consumed was also livestock, particularly cattle (Table 2).

**Fig 3:**
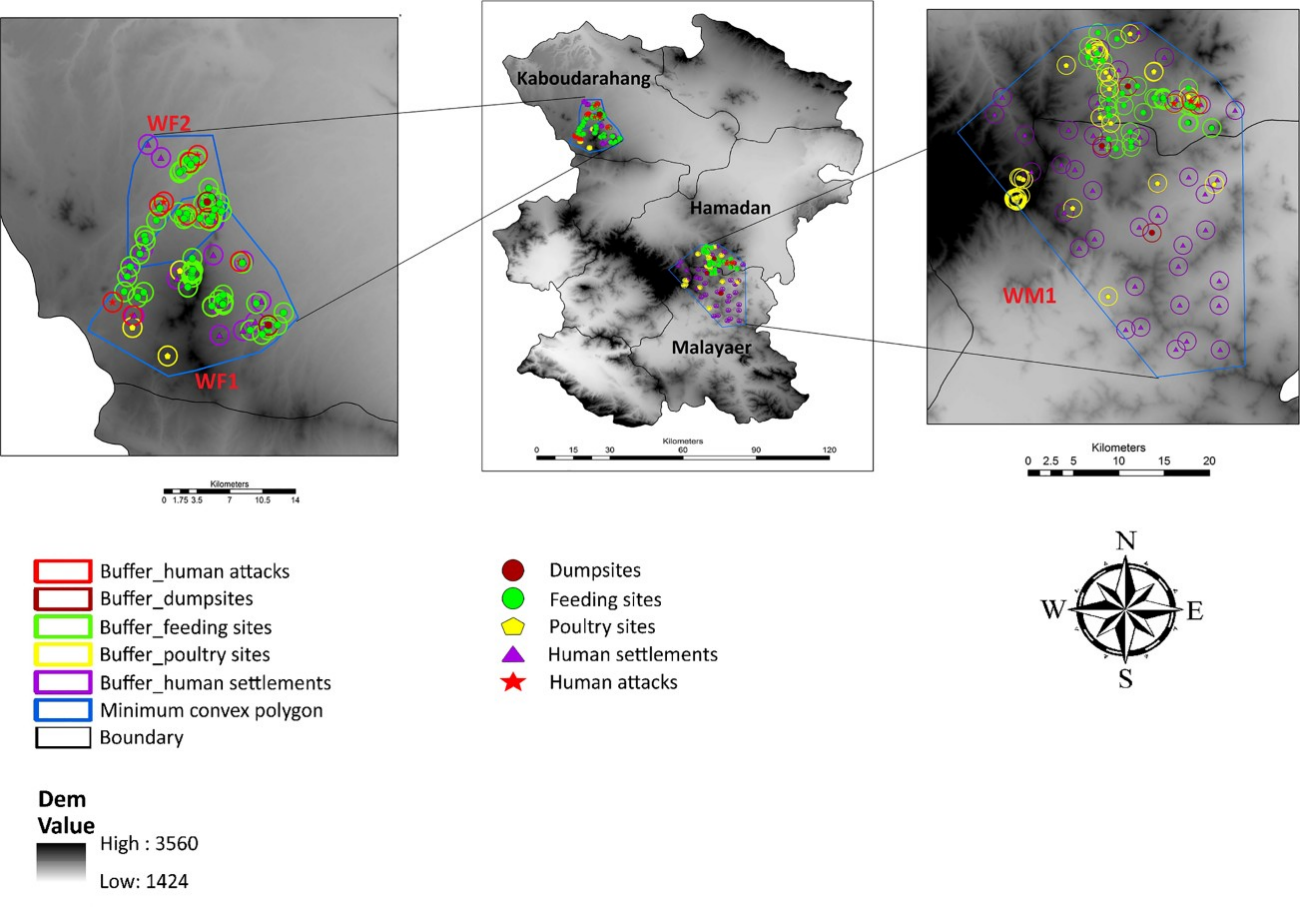
A 1 km circular buffer around every feeding site, human settlement and dumpsites within wolf home ranges (WM1, WF1 and WF2), and the location of wolf attacks on humans recorded since 2001 (DEM map was downloaded from the WorldClim database (www.worldclim.org)).

We found a remarkable spatial overlap between feeding clusters and human settlements (S5 Table); and also between wolf attacks and human settlements (S5 Table) or feeding sites (S5 Table, Fig 3). Wolves tended to feed closer to human settlements than at random locations (Z = -14.36, P< 0.001, n = 312).

### Scat analysis

We collected a total of 110 scat samples; of which only 70 scats were included in subsequent analyses according to the criteria adopted to avoid sampling bias. The number of scat samples within each wolf's home range was 20 (WF1), 30 (WM1), and 20 (WF2). We assumed that the majority of these scats were produced by the collared wolves or other members of their packs. Scat analysis showed that livestock (sheep and cattle) (30.7%), garbage (21.2%), poultry (14.3%), and European hares (13.7%) were the most frequent items found in the diet of wolves (Table 3). Other identified prey species included dog, red fox, yellow ground squirrel (*Spermophilus fulvus*), and small rodents (Table 3).

**Table 3:** Prey items identified in wolf scats.

Food items	N. of prey items	Occurrence in scats	Prey items occur
Livestock (sheep)	35	50	18.5
Livestock (cattle)	23	32.8	12.2
Dog	5	7.1	2.6
Red fox	2	2.8	1.0
European hare	26	37.1	13.7
Yellow ground squirrel	20	28.6	10.6
Small rodents	11	15.7	5.8
Poultry	27	38.6	14.3
Garbage (i.e., plastic bag)	40	57.1	21.2
Total	189	269.6	100

Small prey species (< 2kg), such as yellow ground squirrel and small rodents, were identified in 31 scats (44.3%). None of these prey items were found using GPS clusters. Garbage (i.e., plastic) and poultry were not detected using GPS clusters, probably because wolves invest a very small amount of time in consuming these items. Still, despite these differences, based on relative biomass, livestock (69.3), European hare (22.7), dog (6.20), and red fox (1.7), were the most consumed food items in the scats (Table 4), being the main prey items similar to the ones observed using GPS clusters.

**Table 4:** Relative biomass consumed by wolves in Hamadan Province using scats. Scat samples were analyzed based on occurrence of prey items relative to all prey items identified.

Prey	Estimated weight of prey (kg)	Correction factor (kg/scat)	Prey items occurrence	Total biomass consumed (kg)	Relative biomass consumed (kg)
Livestock (sheep)	25	0.639	18.5	11.8	16.9
Livestock (cattle)	450	4.039	12.2	49.2	70.7
Dog	28	0.663	2.6	1.7	2.5
Red fox	5	0.479	1.0	0.5	0.7
European Hare	3.5	0.467	13.7	6.4	9.2
Total	61.5	6.287	48	69.6	100

We detected significant differences between the feeding behavior of wolves estimated using GPS clusters and scats regarding wolf dietary composition (G_2_ = 4.55, P = 0.02). The results of scat analysis are reported for wolf home range in S6 and S7 Tables.

## Discussion

Wolves in our study area showed an opportunistic dietary behavior feeding on a broad range of food items but with a strong dependence on anthropogenic resources, a finding similar to other areas with low abundance of wild prey [25, 57]. Presumably because such resources are distributed unevenly and concentrated near areas of higher human activity, most of the feeding clusters analyzed were close to human settlements, farms and dumpsites, where organic refuses are disposed by humans. In human-dominated landscapes of western Iran, as a consequence of wild prey depletion and the fact that livestock is generally guarded by sheepherders–reducing the vulnerability of the flocks to wolf attacks-, wolves also depend on alternative anthropogenic sources of food, including garbage if accessible [11,14, 25, 28,38, 57]. Under this scenario, where it may be difficult for them to kill livestock frequently, wolves seem to act mainly as scavengers, with 55% of feeding events being classified as scavenging events.

This scenario may be relatively common across different human-dominated landscapes of Asia, considering the large amount of garbage and domestic animal species in the diet of Asian wolves [11]. For instance, in Yazd province (central Iran), where there is a moderately low abundance of wild prey, grey wolves fed mostly on farmed chicken, domestic goat and garbage [28]. Previous field observations in the Middle East had previously shown a dependence of this species on anthropogenic food sources [27, 28, 58, 59].

Livestock comprised the highest proportion of biomass consumed by wolves in this area, followed by European hare. In Hamadan Province, by disposing of waste and carcasses of domestic animals near human settlements, local communities may be attracting facultative scavengers, such as wolves, to the proximity of human settlements [11,60–61]. In fact, our spatial analyses confirm this pattern. Wolves tend to feed closer to human settlements than by random, which could be a factor affecting the likelihood of an encounter between wolves and humans. In a study conducted by Krithivasan et al., 2009 in India, the most commonly found prey in wolf scats were goat, followed by chicken. In India, it has been documented that in areas with a reasonable abundance of native wild prey, wolf depredation on livestock was much less frequent [57]. Nevertheless, in a completely human-dominated landscape similar to our study area, lack of wild habitats and wild prey is dominant and reintroduction of wild prey is faced with serious difficulties as most habitats for wild prey have been severely degraded and habitat restoration would not be feasible.

The study area holds a large number of poultry farms that illegally dump dead animal carcasses in their surroundings. In this line, we found garbage and poultry in the 58% and 39% of the scats, respectively, suggesting that this food might have been obtained as carrion at disposal sites or around poultry farms in the surrounding villages. Organic waste is known to become a substantial food source for wolves in scarcity of alternative prey [16]. In a study by Capitani et al., 2016 on the feeding ecology of wolves using scat analysis in Kars province, north-eastern Turkey, it was shown that the greatest part of the biomass intake for wolves consisted of livestock.

A similar study in central Iran (Ghamishlou Wildlife Refuge) showed that the high proportion of livestock in wolf scats was related to scavenging behavior rather than predation, as disease was an important mortality factor in local herds [27]. The same is probably true for our study area, where we found that most livestock consumed by collared wolves originated from diseased carcasses abandoned by local sheepherders. In the absence of wild prey, open dumping of livestock carcasses can partly help scavenging wolves to persist [62], which may also influence on the risk of depredation on livestock [28,47].

The estimated frequency of prey consumed by wolves differed significantly between GPS clusters and scats. Similarly to other studies, this study showed that analyzing scat samples reveals a greater diversity of prey species, including small-bodied prey, and therefore it can provide a complementary picture of the wolf diet, whereas kill site investigation is biased toward medium to large-bodied prey species [46,63]. Nonetheless, GPS cluster analysis provide valuable information for livestock damage assessment and management, allowing to discriminate scavenging from predation events [51], otherwise impossible from simple scat analysis. Also, unlike scats, GPS cluster analysis allows a spatial analysis of diet at fine scales. Using this approach, researchers can gather valuable information about the prey’s sex, age and condition [53,64], which cannot be obtained by simply employing the scat analysis. Our results in relation to scat analyses should be interpreted with some caution considering the low sample size, which might have caused biased conclusions.

## Conclusion

In Hamadan province, Wild prey was rarely found in the diet of wolves, being comprised by small to medium mammal species. Hamadan is then a good example of a native prey depleted area where wolves may have persisted shifting their diet base to anthropogenic food sources, mainly found at the vicinity of human settlements. In a similar study conducted by Tourani et al. [2014], foraging on poultry dumps by wolves is also reported, in this case being the main food item. Poultry farms are mandated to burn chicken carcasses within their facilities, but illegal dumping is widespread in this area. Access of wildlife to waste can exacerbate negative interactions between humans and wildlife [65].

We strongly recommend that sheep herders and local communities avoid abandoning animal carcasses near their pastures, houses, and farmlands to minimize improper disposal of livestock. Appropriate management of illegal dumping of animal carcasses and garbage dumps would reduce the chances of human-wolf encounters in this scenario [28,66–67]. Previous studies on areas where anthropogenic resources were essential to wolves’ persistence [14,25, 56] have also suggested that to reduce the risk of encounters between wolves and humans, effective management of dumpsites and carcass disposal may be an important intervention in those areas. We encourage managers to undertake at the same time preliminary research and delineate actions aiming not only to reduce the accessibility of wolves to animal carcasses and organic waste, but also to restore the unbalanced ecosystem in the mid-term, improving native prey base wherever suitable habitat and Social context remains. Long-term assessment of wolf ecology in Hamadan province is certainly needed to propose optimal solutions to reduce human-wolf conflict in this part of Iran.

## Supporting information

S1 Table Mean prey body weight of each prey (Kg) obtained from the Atlas of Mammals of Iran. (DOCX)

S2 Table WF1 feeding remains located using clusters of GPS locations. (DOCX)

S3 Table WM1 feeding remains located using clusters of GPS locations. (DOCX)

S4 Table WF2 feeding remains located using clusters of GPS locations. (DOCX)

S5 Table Percentage of spatial overlap between feeding sites, wolf attacks, dumpsites and human settlements according to a 1 km circular buffer around each event within home ranges of each tracked wolf. (DOCX)

S6 Table Composition of wolves' diet in Hamadan province. Scats analyzed by occurrence of prey items relative to total prey items.
(DOCX)

S7 Table Composition of wolves' diet based on biomass consumed. (DOCX)
