# Supplementary material for: Anthropogenic food resources sustain wolves in conflict scenarios of Western Iran
Source: PLoS One. 2019 Jun 17;14(6):e0218345. doi: 10.1371/journal.pone.0218345 (PMC6576759; doi:10.1371/journal.pone.0218345)
Supplement: S1 Table — (DOCX) [file pone.0218345.s001.docx]

**S1 Table. Mean prey body weight of each prey (Kg) obtained from the Atlas of Mammals of Iran.**

| **Prey type** | **Mean weight of prey (kg)** |
| --- | --- |
| Livestock (sheep) | 25 |
| Livestock (cattle) | 450 |
| European hare | 3.5 |
| Golden jackal | 11 |
| Red fox | 5 |
| Dog | 28 |
