# Supplementary material for: Anthropogenic food resources sustain wolves in conflict scenarios of Western Iran
Source: PLoS One. 2019 Jun 17;14(6):e0218345. doi: 10.1371/journal.pone.0218345 (PMC6576759; doi:10.1371/journal.pone.0218345)
Supplement: S2 Table — (DOCX) [file pone.0218345.s002.docx]

**S2 Table. WF1 feeding remains located using clusters of GPS locations.**

|  | | | | **predation (40)** | | **scavenging (70)** | | | |
| --- | --- | --- | --- | --- | --- | --- | --- | --- | --- |
| **Prey** | **No. of kills** | **% of kills** | **Biomass consumed (kg)** | | **Biomass consumed as % of all kill sites** | **No. of carcass eaten** | **% of carcass eaten** | **Biomass consumed (kg)** | **Biomass consumed as % of all kill sites** |
| Livestock (domestic sheep) | 28 | 70 | 700 | | 88.5 | 52 | 74.3 | 1300 | 26.1 |
| cattle | 0 | 0 | 0 | | 0 | 8 | 11.4 | 3600 | 72.3 |
| European Hare | 10 | 16.6 | 35 | | 4.3 | 0 | 0 | 0 | 0 |
| Golden jackal | 0 | 0 | 0 | | 0 | 5 | 7.1 | 55 | 1.1 |
| Red fox | 0 | 0 | 0 | | 0 | 5 | 7.1 | 25 | 0.5 |
| Dog | 2 | 3.3 | 56 | | 6.9 | 0 | 0 | 0 | 0 |
| Total | 40 | 89.9 | 791 | | 99.5 | 70 | 99.9 | 4980 | 100 |
