# Supplementary material for: Anthropogenic food resources sustain wolves in conflict scenarios of Western Iran
Source: PLoS One. 2019 Jun 17;14(6):e0218345. doi: 10.1371/journal.pone.0218345 (PMC6576759; doi:10.1371/journal.pone.0218345)
Supplement: S3 Table — (DOCX) [file pone.0218345.s003.docx]

**S3 Table. WM1 feeding remains located using clusters of GPS locations.**

|  |  |  | **depredation (90)** | | | **scavenging (95)** | | | |
| --- | --- | --- | --- | --- | --- | --- | --- | --- | --- |
| **Prey** | **Estimated mean weight of prey (kg)** | **No. of kills** | **% of kills** | **Biomass consumed (kg)** | **Biomass consumed as % of all kill sites** | **No. of carcass eaten** | **% of carcass eaten** | **Biomass consumed (kg)** | **Biomass consumed as % of all kill sites** |
| Livestock (domestic sheep) | 25 | 68 | 75.5 | 1700 | 91.3 | 61 | 64.2 | 1525 | 21.4 |
| cattle | 450 | 0 | 0 | 0 | 0 | 12 | 12.6 | 5400 | 76.1 |
| European Hare | 3.5 | 16 | 17.7 | 56 | 3 | 0 | 0 | 0 | 0 |
| Golden jackal | 11 | 1 | 1. 1 | 11 | 0.6 | 10 | 10.5 | 110 | 1.5 |
| Red fox | 5 | 2 | 2.2 | 10 | 0.5 | 12 | 12.6 | 60 | 0.9 |
| Dog | 28 | 3 | 3.3 | 84 | 4.5 | 0 | 0 | 0 | 0 |
| Total | 547.5 | 90 | 100 | 1861 | 100 | 95 | 99.9 | 7095 | 100 |
