# Supplementary material for: Anthropogenic food resources sustain wolves in conflict scenarios of Western Iran
Source: PLoS One. 2019 Jun 17;14(6):e0218345. doi: 10.1371/journal.pone.0218345 (PMC6576759; doi:10.1371/journal.pone.0218345)
Supplement: S4 Table — (DOCX) [file pone.0218345.s004.docx]

**S4 Table. WF2 feeding remains located using clusters of GPS locations.**

|  | **depredation (12)** | | | | | | **scavenging (5)** | | |
| --- | --- | --- | --- | --- | --- | --- | --- | --- | --- |
| **Prey** | **No. of kills** | **% of kills** | **Biomass consumed (kg)** | **Biomass consumed as % of all kill sites** | **No. of carcass eaten** | **% of carcass eaten** | | **Biomass consumed (kg)** | **Biomass consumed as % of all kill sites** |
| Livestock (domestic sheep) | 10 | 83. 3 | 250 | 97.2 | 2 | 40 | | 50 | 9.6 |
| cattle | 0 | 0 | 0 | 0 | 1 | 20 | | 450 | 87.2 |
| European Hare | 2 | 13.3 | 7 | 2.7 | 0 | 0 | | 0 | 0 |
| Golden jackal | 0 | 0 | 0 | 0 | 1 | 20 | | 11 | 2.1 |
| Red fox | 0 | 0 | 0 | 0 | 1 | 20 | | 5 | 0.9 |
| Total | 12 | 96.6 | 257 | 100 | 5 | 100 | | 516 | 100 |
