# Supplementary material for: Anthropogenic food resources sustain wolves in conflict scenarios of Western Iran
Source: PLoS One. 2019 Jun 17;14(6):e0218345. doi: 10.1371/journal.pone.0218345 (PMC6576759; doi:10.1371/journal.pone.0218345)
Supplement: S5 Table — (DOCX) [file pone.0218345.s005.docx]

**S5­ Table. Percentage of spatial overlap between feeding sites, wolf attacks, dumpsites and human settlements according to a 1 km circular buffer around each event within home ranges of each tracked wolf.**

|  | | **Feeding clusters** | **Wolf attacks** | **Dump sites** | **Human settlement** |
| --- | --- | --- | --- | --- | --- |
| WM1 | Feeding clusters | 100 | 50 | 45 | 58 |
|  | Wolf attacks | 50 | 100 | 0 | 50 |
|  | Dump sites | 45 | 0 | 100 | 30 |
|  | Human settlement | 58 | 50 | 30 | 100 |
| WF1 | Feeding clusters | 100 | 20 | 55 | 60 |
|  | Wolf attacks | 20 | 100 | 10 | 90 |
|  | Dump sites | 55 | 10 | 100 | 35 |
|  | Human settlement | 60 | 90 | 35 | 100 |
| WF2 | Feeding clusters | 100 | 15 | 52 | 55 |
|  | Wolf attacks | 15 | 100 | 12 | 85 |
|  | Dump sites | 52 | 12 | 100 | 38 |
|  | Human settlement | 55 | 85 | 38 | 100 |
