# Supplementary material for: Anthropogenic food resources sustain wolves in conflict scenarios of Western Iran
Source: PLoS One. 2019 Jun 17;14(6):e0218345. doi: 10.1371/journal.pone.0218345 (PMC6576759; doi:10.1371/journal.pone.0218345)
Supplement: S6 Table — Scats analyzed by occurrence of prey items relative to total prey items. (DOCX) [file pone.0218345.s006.docx]

**S6 Table. Composition of wolves' diet in Hamadan province. Scats analyzed by occurrence of prey items relative to total prey items.**

| **WF2(n=20)** | | | **WF1(n=20)** | | | **WM1(n=30)** | | |  |
| --- | --- | --- | --- | --- | --- | --- | --- | --- | --- |
| **Prey items occur** | **Scats occur** | **No. of prey items** | **Prey items occur** | **Scats occur** | **No. of prey items** | **Prey items occur** | **Scats occur** | **No. of prey items** | **Prey** |
| 16.9 | 50 | 10 | 19.2 | 50 | 10 | 19.2 | 50 | 15 | Livestock (sheep) |
| 10.1 | 30 | 6 | 13.4 | 35 | 7 | 12.8 | 33.33 | 10 | Livestock (Cattle) |
| 0 | 0 | 0 | 3.8 | 10 | 2 | 3.8 | 10 | 3 | Dog |
| 0 | 0 | 0 | 0 | 0 | 0 | 2.5 | 6.6 | 2 | Red fox |
| 6.7 | 20 | 4 | 3.8 | 10 | 2 | 25.6 | 66.6 | 20 | European Hare |
| 16.9 | 50 | 10 | 19.2 | 50 | 10 | 0 | 0 | 0 | Yellow Ground Squirrel |
| 10.1 | 30 | 6 | 0 | 0 | 0 | 6.4 | 16.6 | 5 | Small Rodents |
| 16.9 | 50 | 10 | 17.3 | 45 | 9 | 10.2 | 26.6 | 8 | Poultry |
| 22 | 65 | 13 | 23.1 | 60 | 12 | 19.2 | 50 | 15 | Garbage (i.e., plastic bag) |
| 99.9 | 295 | 59 | 99.9 | 260 | 52 | 99.9 | 259.9 | 78 | Total |
