# Supplementary material for: Anthropogenic food resources sustain wolves in conflict scenarios of Western Iran
Source: PLoS One. 2019 Jun 17;14(6):e0218345. doi: 10.1371/journal.pone.0218345 (PMC6576759; doi:10.1371/journal.pone.0218345)
Supplement: S7 Table — (DOCX) [file pone.0218345.s007.docx]

**S7 Table. Composition of wolves' diet based on biomass consumed.**

| **WF2** | | | **WF1** | | | **WM1** | | | | | |
| --- | --- | --- | --- | --- | --- | --- | --- | --- | --- | --- | --- |
| **Relative biomass consumed (kg)** | **Total biomass consumed (kg)** | **Prey items occur** | **Relative biomass consumed (kg)** | **Total biomass consumed (kg)** | **Prey items occur** | **Relative biomass consumed (kg)** | **Total biomass consumed (kg)** | **Prey items occur** | **Correction factor (kg/scat)** | **Est. wt of prey (kg)** | **Prey** |
| 19.9 | 10.7 | 16.9 | 17.3 | 12.2 | 19.2 | 15.4 | 12.2 | 19.2 | 0.639 | 25 | Livestock (sheep) |
| 74.5 | 40.7 | 10.1 | 76.7 | 54.1 | 13.4 | 65 | 51.6 | 12.8 | 4.039 | 450 | Livestock (Cattle) |
| 0 | 0 | 0 | 3.6 | 2.5 | 3.8 | 3.1 | 2.5 | 3.8 | 0.663 | 28 | Dog |
| 0 | 0 | 0 | 0 | 0 | 0 | 1.5 | 1.2 | 2.5 | 0.479 | 5 | Red fox |
| 5.6 | 3.1 | 6.7 | 2.4 | 1.7 | 3.8 | 15 | 11.9 | 25.6 | 0.467 | 3.5 | European Hare |
| 100 | 54.68 | 33.7 | 100 | 70.58 | 40.2 | 100 | 79.55 | 63.9 | 6.287 | 511.5 | Total |
